# Clinical characteristics, prognostic indicators, and survival outcomes in intravascular lymphoma: Mayo Clinic experience (2003–2018)

**DOI:** 10.1002/ajh.26635

**Published:** 2022-07-06

**Authors:** Karan Seegobin, Zhuo Li, Muhamad Alhaj Moustafa, Umair Majeed, Jing Wang, Liuyan Jiang, Justin Kuhlman, David Menke, Ke Li, Mohamed A. Kharfan‐Dabaja, Ernesto Ayala, Madiha Iqbal, Grzegorz S. Nowakowski, Thomas M. Habermann, Thomas E. Witzig, Patrick Johnston, Carrie Thompson, Stephen Ansell, Han W. Tun

**Affiliations:** ^1^ Department of Haematology and Medical Oncology Mayo Clinic Jacksonville Florida USA; ^2^ Department of Biomedical Statistics and Informatics Mayo Clinic Jacksonville Florida USA; ^3^ Department of Internal Medicine Mayo Clinic Jacksonville Florida USA; ^4^ Department of Pathology Mayo Clinic Jacksonville Florida USA; ^5^ Department of Haematology and Medical Oncology Mayo Clinic Rochester Minnesota USA

## Abstract

Intravascular lymphoma (IVL) is a rare extranodal non‐Hodgkin lymphoma. We performed a retrospective analysis of 55 IVL patients who were treated at our institution 2003–2018. Median age at diagnosis was 68 years, and 64% were males. The most frequent presenting symptoms were skin rash 43% and weight loss 30%. MRI brain on IVL patients with CNS involvement (CNS‐IVL) showed multifocal involvement in 76% (13/17). 89% (17/19) of non‐CNS‐IVL patients with abnormal FDG‐PET had biopsy of an avid lesion resulting in definitive diagnosis. The top diagnostic biopsy site was the bone marrow (45%). 56% had multiorgan involvement. Based on CNS involvement, 36.5% (20/55) had CNS‐IVL and 63.5% (35/55) had non‐CNS‐IVL. CNS‐IVL group consists of clinically isolated CNS involvement (CNS‐only IVL) (22%;12/55) and mixed clinical CNS and peripheral site involvement (M‐IVL) (14.5%; 8/55). Non‐CNS‐IVL group consists of clinically isolated skin involvement (skin‐only IVL) (9%; 5/55) and peripheral IVL with or without skin involvement (P‐IVL); (54.5%; 30/55). Skin involvement was predominantly in the lower extremities. Pathologically, 89% (48/54) were B‐cell IVL. Rituximab + high‐dose methotrexate‐based regimen were used in 75% (12/16) of CNS‐IVL patients and RCHOP in 60% (17/28) of non‐CNS‐IVL patients. Estimated 5‐year progression free survival (PFS) and overall survival (OS) for the entire cohort were 38.6% and 52%, respectively. Skin‐only IVL was associated with excellent survival. Platelet count <150x10^9^/L, age > 60Y, and treatment without Rituximab were poor prognostic factors. Further research is necessary to identify novel therapies.

## 1 INTRODUCTION

IVL is a rare aggressive non‐Hodgkin lymphoma which was first described by Pfleger and Tappeiner in 1959.^1^ The WHO classification of hematopoietic tumors defines IVLBCL as an extranodal diffuse LBCL characterized by the presence of neoplastic lymphocytes only in the lumina of small vessels, particularly in the capillaries.^2^ The incidence rate reported in 2017 was 0.095/100 000 population (~1/10 00 000) and between the years of 2000 and 2013, 344 cases were identified.^3^ The blood vessel lumen is not only the disease vehicle but also its site of replication; the tumor cells express molecules that facilitate cell migration and adhesion to the endothelium, but lack those involved in extravasation.^4^ While predominantly intravascular, IVL with extravascular component has also been described.^5^ The peculiar affinity of lymphoma cells to the intravascular space has been linked to the absence of CD29 (b1 integrin) and CD54 (ICAM‐1) surface ligands, which may disable them from diapedesis across the endothelium.^6^

IVL usually affects elderly patients, commonly presenting in the sixth or seventh decades of life.^7^, ^8^, ^9^ Central nervous system (CNS) and skin are primarily involved. Liver, spleen, lymph nodes, and bone marrow are relatively spared until later in the disease course.^10^, ^11^ Presentation with tumor or lymphadenopathy is not common in IVL.^3^ Clinical manifestations can range from mild symptoms such as fever, pain or organ‐specific local symptoms, and combination of B symptoms to rapidly progressing multiorgan failure.^12^ Due to vascular obstruction and thrombosis, there can be ischemic tissue damage with attendant clinical manifestations such as stroke.^10^ Hemophagocytosis can be seen in IVL and is more common in Asian patients.

Due to the low incidence of IVL, our understanding of the characteristics, management, and outcomes of IVL relies on small retrospective studies. We report clinical characteristics and survival outcomes of IVL in the setting of a large multi‐center academic setting.

## 2 METHODS

We conducted a retrospective single‐institution multi‐center analysis of IVL which was approved by the Institutional Review Board. We identified patients with IVL managed at Mayo Clinic Cancer Center between January 2003 and December 2018. Demographic, clinical, radiologic, pathologic, and therapeutic data were extracted from the clinical records. Categorical variables were summarized as frequency (percentage) and continuous variables were reported as median (range) and mean (standard deviation). Data were compared between CNS and non‐CNS groups using Wilcoxon rank sum test for continuous variables and Fisher's exact test for categorical variables. The 1‐, 3‐, and 5‐year overall survival (OS) and progression‐free survival (PFS) were estimated, and the corresponding survival curves were drawn using Kaplan–Meier methods. Log‐rank test was used to compare OS and PFS between patients with and without a risk factor. All tests were two‐sided with p value of <0.05 considered statistically significant. The analysis was done using R4.0.3, and Blue‐Sky Statistics Software.

## 3 RESULTS

### 3.1 Demographics, clinical characteristics, radiological, laboratory, and pathologic findings (Figures 1, 2 and Tables S1‐S6, S8)

**FIGURE 1:**
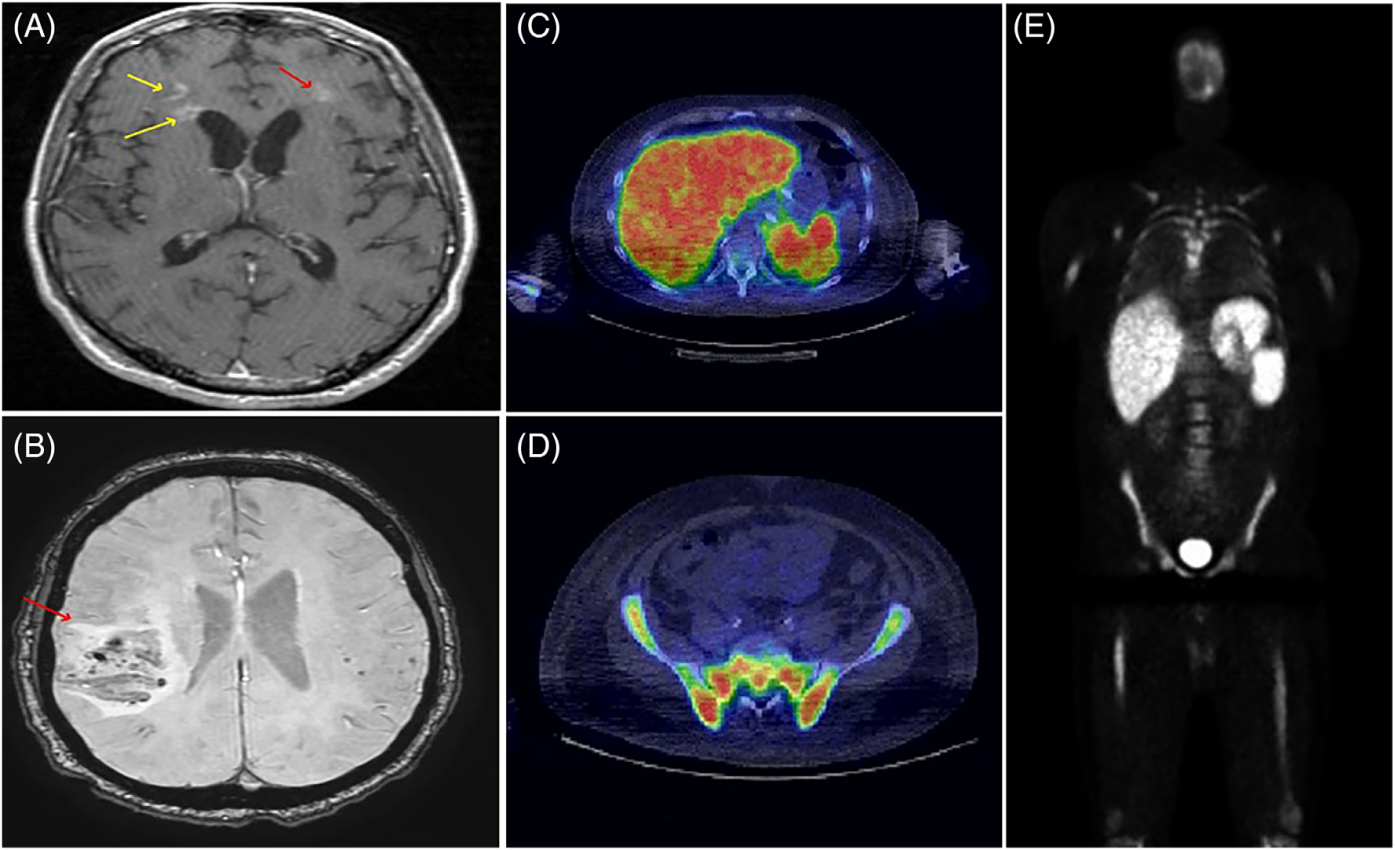
(A) Axial T1 MRI brain showing multifocal enhancing lesions in bilateral frontal lobes. (B) Axial SWI MRI brain showing mass lesion in the right parietal region with surrounding mass effect and compression of the right ventricle. (C–E) PET CT with increased FDG avidity in liver, spleen, and bone marrow with SUV 12.4 in bone marrow [Color figure can be viewed at wileyonlinelibrary.com]

**FIGURE 2:**
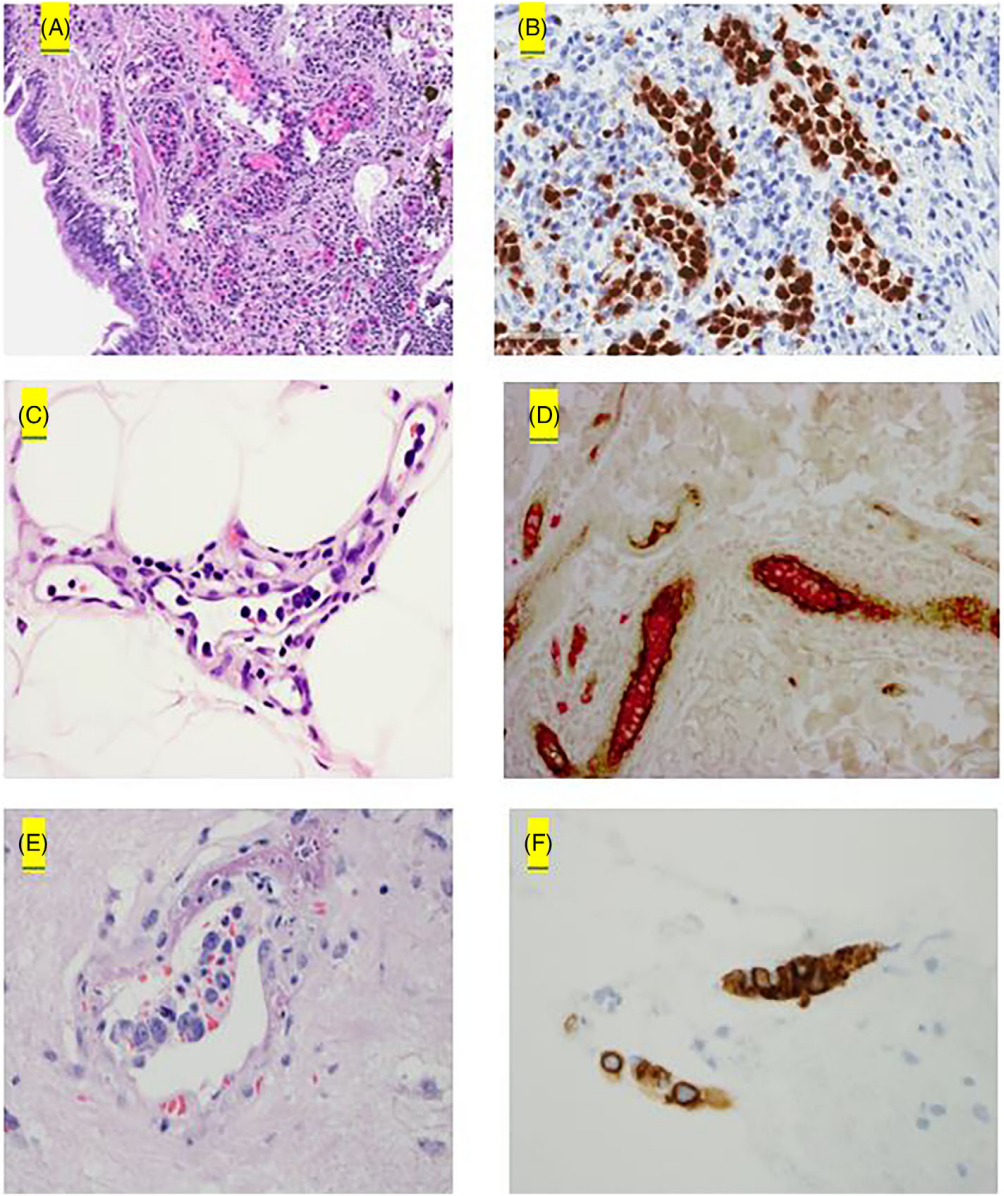
Pathologic findings in IVL. (A, B) IVL in the lung is highlighted by PAX5. (C, D) IVL in the skin with lymphoma cells positive for CD20 (red) within the vessels and vessel wall highlighted by Factor VIII (brown). (E, F) CNS‐IVL with lymphoma cells the IVL cells positive for CD20 [Color figure can be viewed at wileyonlinelibrary.com]

The median age at diagnosis was 68 (range 40–85) years and 64% were males. Racial distribution showed 47 Whites, 1 Black, 1 Asian, 1 Other, and 5 were undisclosed in the medical records. ECOG performance status was ≤2 in 83% pts at the time of diagnosis. ECOG of all patients with Skin‐only IVL was ≤1 at diagnosis. The median time from symptom onset to diagnosis was 90 days (range 7–596), and median time from admission to our facility to diagnosis was 10 days (range 1–386) (Figures 1, 2 and Tables S1‐S6, S8).

In the entire cohort, the common presenting symptoms include skin rash 43% (13/30), weight loss 30% (15/49), fatigue 28% (14/49), extremity weakness/gait impairment 22% (11/49), night sweats 21% (8/38), fever 21% (8/38), visual disturbances 21% (4/19), shortness of breath 20% (10/49), and memory disturbances/confusion 20% (10/49). On exam positive clinical signs included splenomegaly 38.4% (15/39), skin rash 23% (7/30), altered mental status 20% (10/49), lymphadenopathy 13% (4/30), petechiae/bruising 10% (3/30), pedal edema 10% (3/30), and hepatomegaly 7.6% (3/39).

In patients with CNS‐IVL, the common clinical features at presentation were bowel/bladder incontinence (45%; 5/11), extremity weakness/gait impairment (42%; 8/19), fever (38%; 3/8), memory disturbances/confusion (36%; 7/19), paraesthesia of hands/feet (26%; 5/19), fatigue (26%; 5/19), night sweats (25%; 2/8), and visual disturbances (21%; 4/19). In those with non‐CNS‐IVL, weight loss (40%; 12/30), skin rash (43%; 13/30), fatigue (30%; 9/30), shortness of breath (20%; 6/30), and night sweats (20%; 6/30) were the most common.

At the time of diagnosis, 91% (43/47) were anemic with mean hemoglobin of 9.6 g/dL, 30% (14/46) were leukopenic with mean total white blood cell count (WCC) of 2.5 × 10^9^/L, 45% (21/47) were thrombocytopenic with the mean platelet count of 59.4 × 10^9^/L, and 68% (17/25) had elevated ESR with the mean value of 69.1 mm/1H. In those with T‐cell IVL, (3/4) were anemic with mean hemoglobin of 10 g/dL, (1/4) were leukopenic (with mean WCC 1.3 × 10^9^/L), and (1/4) were thrombocytopenic (with mean platelet count 15 × 10^9^/L). The patient with NK‐cell IVL was anemic (Hb = 7.7 g/dL) and thrombocytopenic with platelet count of 12 × 10^9^/L at presentation. The median LDH was 559 U/L in entire cohort (range 123–6800).

MRI was not available in the records for three patients with CNS IVL, two of which were diagnosed by autopsy. Fifteen patients had abnormal brain MRI, one with abnormal thoracic spinal cord and brain MRI, and one patient with abnormal thoracic spinal cord with normal brain. The thoracic spinal cord imaging showed multifocal abnormal patchy signal consistent with lymphoma in these two patients. MRI imaging showed multifocal involvement in 76% (13/17), mass lesions in 23% (4/17), and infarcts in 29% (5/17). It is noteworthy to report that in 15/17 MRI reports, there were no comment of lymphoma as a possible differential diagnosis in the radiology reports. In the two cases where lymphoma was part of the differential in the report, multifocal white matter abnormalities, diffusion restriction, patchy hyperintense lesions, and increased T2 signal were features mentioned in the report; however, other differentials were also included in these reports such as vasculitis, demyelinating process, Susac's syndrome, chronic infectious process, neurosarcoidosis, and other granulomatous disease. Biopsy of a suspicious MRI lesion ultimately led to the diagnosis of IVL.

Regarding PET findings in the non‐CNS‐IVL patients, 27 patients had PET scans at diagnosis; 70% (19/27) had abnormal PET scans with 89% of those with abnormal PET scans (17/19) having a biopsy of PET avid lesion that led to the diagnosis of IVL. Diffuse bone marrow uptake was reported on PET imaging in ten patients with diagnostic bone marrow biopsy. Two patients with diagnostic bone marrow biopsy had physiologic uptake reported. Four patients with bone marrow involvement had SUV >10 in the bone marrow. In those with T‐cell IVL, 50% (2/4) had abnormal PET. In one case, the PET was avid in the mediastinal lymph nodes, lung parenchyma nodules, and bone marrow; biopsy of the lung lesion confirmed the diagnosis. In the second case, the PET was avid in the skin and lung nodule; biopsy of the skin led to the diagnosis. In the patient with NK‐cell IVL, the PET scan showed diffuse increased uptake in the bone marrow and spleen. Biopsy of the bone marrow led to the diagnosis.

In the entire cohort, (56%; 31/55) of patients had multiorgan involvement at diagnosis. Brain (32%; 18/55), spleen 27% (15/55), lymph nodes (16%; 9/55), liver (14%; 8/55), and lung (12%; 7/55) were the more common organs involved based on the initial clinical assessment.

In the entire cohort, the top three common diagnostic biopsy sites were bone marrow (BM) 45% (25/55), skin 25% (14/55), and brain 29% (16/55). In non‐CNS‐IVL, the diagnosis was made with biopsies of the following sites: bone marrow 54% (19/35), skin 40% (14/35), lung 14% (5/35), liver 5.7% (2/35), spleen 2.8% (1/35), and omentum 2.8% (1/35); 18 patients had positive biopsy of more than one site.

In those with skin involvement, 35% (5/14) had skin‐only disease. 86% (12/14) patients with cutaneous manifestations had involvement of lower extremities. One patient did not have skin manifestations on exam and had positive diagnostic radom skin biopsies. Bilateral lower extremity involvement was described in (5/8). Multifocal skin involvement including lower extremity was seen in (3/8).

In patients with non‐CNS IVL, 97% (36/37) had the presence of at least one cytopenia; in this subset of patients with cytopenias, the bone marrow was involved with IVL in 68% (23/34) cases. Of the 25 patients with bone marrow involvement, 68% (17/25) had ≤20% bone marrow involement, one patient had 40% involvement, and two patients had ≥50% involvement. The degree of bone marrow involvement could not be determined in the other five patients. In those with T cell IVL, the diagnosis was made with skin biopsy (4/5), bone marrow biopsy (2/5), and lung biopsy (1/5). The patient with NK cell IVL was diagnosed via bone marrow biopsy. In patients with CNS‐IVL, (8/10) had the presence of at least one cytopenia.

Regarding lymphocyte lineage, 89% (48/54) of patients had B‐cell IVL, 9% (5/54) had T‐cell IVL, one patient had NK‐cell IVL, and one patient had unknown lymphocyte lineage.

Pathologically, B‐cell IVL showed expression of BCL2, BCL6, MYC, and MUM1 in 100% (19/19), 61.9% (13/21), 71.4% (5/7), and 77% (17/22) of patients, respectively. 17% (1/6) with B cell IVL had BCL6 FISH rearrangement. There was no rearrangement of BCL2 or MYC on FISH analysis (0/6).

In those with T‐cell IVL, IHC showed BCL2 in (1/1). There were no patients with BCL6 (0/1) or MUM1 (0/1) IHC expression; furthermore. Pathologic IHC and FISH results were not available for several patients with non‐B cell IVL. Markers for identifying NK cell phenotype included CD45+, CD2+, CD56+, TIA‐1 + and granzyme B+. Markers for T cell phenotype were CD2+, CD3+, CD4+, CD5+, and CD45RO+; TCR gamma/delta+ or TCR alpha/beta+.

We highlight the pathologic findings of IVL in the lung, skin, and CNS with intravascular localization of lymphoma cells in Figure 2.

EBV‐ISH (EBV in situ hybridization) was available for 12 patients, 11 were negative and one positive. The one positive result was seen in a white male patient with non‐CNS B cell‐IVL who was treated with RCHOP.

Based on central nervous system (CNS) involvement, IVL cases can be grouped as follows.

1. IVL with CNS involvement (CNS‐IVL)‐ 36.5% (20/55)

- IVL with clinically isolated CNS involvement (CNS‐only IVL)‐ 22% (12/55)
- IVL with mixed clinical CNS and peripheral site involvement (M‐IVL)‐ 14.5% (8/55)

1. IVL without CNS involvement (non‐CNS‐IVL)‐ 63.5% (35/55)

- IVL with clinically isolated skin involvement (Skin‐only IVL)‐ 9% (5/55)
- IVL with peripheral site involvement with or without skin involvement (P‐IVL)‐ 54.5% (30/55)

Four patients were diagnosed at autopsy. Of those diagnosed at autopsy, there were three males and one female with median age of 70 years (range 48–71). The median time from presentation to diagnosis by autopsy was ten days (range 6–24). Two patients had CNS‐only IVL and two with mixed‐IVL. Two patients with mixed‐IVL had bone marrow involvement. MRI brain was available for two patients, one showed leptomeningeal enhancement, and the other chronic lacunar infarction in the basal ganglia. They had severe thrombocytopenia with three patients having platelets <20 × 10^9^/L.

No significant difference was seen between CNS‐IVL and non‐CNS‐IVL for age at diagnosis, gender, ECOG performance status, time of diagnosis, cell lineage, BCL6 expression, Myc expression, MUM1 expression, platelet count, leucocyte count, and ESR.

Skin involvement was predominantly located in lower extremities unilaterally or bilaterally (86%; 12/14). Lower extremities were involved in all five cases of skin‐ only IVL, with four patients having bilateral involvement.

All five T‐cell IVL cases were males, with a median age at diagnosis of 70 years (range 68–79). ECOG was ≤2 in (3/4) at diagnosis. Clinical features of T‐cell IVL at presentation were skin rash (3/4), weight loss (1/4) and dyspnea (1/4). Skin manifestations were erythematous rash (3/3), superficial erosions (2/3), and plaque‐like lesions (1/3).

The patient with NK‐cell IVL was an 85 year‐old female with ECOG 3 at diagnosis. Her symptoms had been ongoing for about a year prior to diagnosis and included fatigue, easy bruising, weight loss, and subconjunctival hemorrhage.

In our report, among those with lymph node involvement (nine patients), only two had biopsy of their involved lymph node; among these two patients, one had intravascular as well as parenchymal involvement of the lymph node. Among the patients with splenic involvement (15 patients), only one had biopsy of the spleen; this showed both intravascular and parenchymal involvement.

In our cohort, eight patients were CD5 positive, and seven were CD5 negative. CD5 expression was not available in the remaining 40 patients. Twelve patients were CD10‐ve, one patient CD10+, and CD 10 unavailable in two patients. CD5 positivity was seen in 6 B cell‐IVL cases and 2 T cell‐IVL patients. CD5 negativity was seen in 6 B cell‐IVL cases and the NK cell IVL patient. The mean platelet count at diagnosis was higher in patients with CD5 positivity with mean 140 × 10^9^/L (range 25–266) compared with 65 × 10^9^/L (range 6–296) in CD 5 negative cases. 4/8 CD5+ patients were thrombocytopenic at diagnosis, whereas 6/7 CD5 negative cases were thrombocytopenic at diagnosis.

### 3.2 Therapeutic interventions (Table S1)

The most common first‐line regimens were rituximab + HD‐MTX‐based chemoimmunotherapy (CIT) (75%; 12/16) in CNS‐IVL and RCHOP‐based CIT (60%; 17/28) in non‐CNS‐IVL. Other regimens used in non‐CNS‐IVL are CHOP (14%; 4/28), R‐Hyper‐CVAD (7%; 2/28), cyclophosphamide/doxorubicin/etoposide (3%; 1/28), R‐CHP (3%; 1/28), nitrogen mustard/rituximab (3%; 1/28), ProMACE/CytaBOM (3%; 1/28), and rituximab/mechlorethamine/methylprednisone (3%; 1/28). In those with CNS‐IVL who did not receive HDMTX‐based regimen, three patients received CHOP‐like regimen and one received rituximab/methylprednisolone/mechlorethamine. In the cohort of patients with B‐cell IVL that received treatment, 10% (4/39) did not receive rituximab, whereas 89.7% (35/39) received rituximab (Table S1).

19% (9/47) of patients received autologous stem cell transplant (ASCT); 44% (4/9) pts were transplanted in first complete remission (CR1); and 56% (5/9) were transplanted after the first relapse.

In the non‐CNS‐IVL group, 50% (14/28) received CNS prophylaxis. HD‐MTX was used in 85% (12/14) of patients. 8% (3/35) of non‐CNS‐IVL patients had relapse in CNS. Two out of the three patients who had CNS relapse received CNS prophylaxis. The median time to CNS relapse was 9 months.

In those with T‐cell IVL, CHOP chemotherapy was used in all (4/4). (1/4) received CNS prophylaxis with HD‐MTX, and (0/4) had CNS relapse. (1/4) had progression of disease after first line treatment and received salvage ICE chemotherapy followed by autologous stem cell transplant.

Among the five patients with skin‐only IVL, three were B cell‐IVL and two were T cell‐IVL. Therapy received was not available for two patients (1 B cell and 1 T cell‐IVL). Two patients with B cell‐ IVL received RCHOP and RCHOP with HD‐MTX, respectively. HD‐MTX was added in in the second case due to delay in diagnosis and concern for high risk of relapse by the managing physician. The last patient with T cell‐IVL received CHOP.

### 3.3 Survival outcomes and prognostic indicators (Figures 3 and 4, Tables S7‐S9, Figures S1‐S4)

**FIGURE 3:**
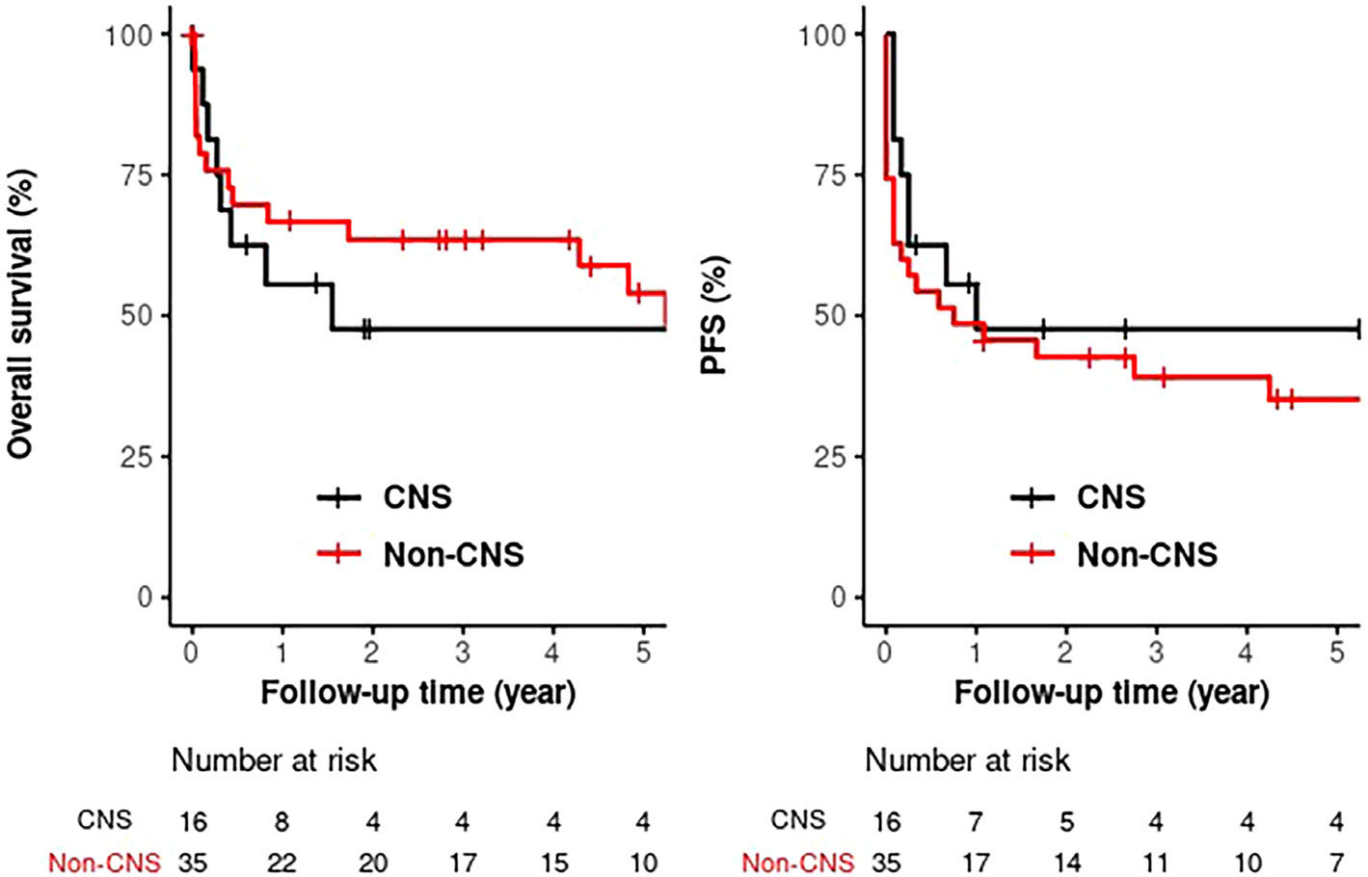
Kaplan–Meier survival curves showing mOS (*p* = .81) and mPFS (*p* = .33) of CNS IVL versus non‐CNS IVL [Color figure can be viewed at wileyonlinelibrary.com]

**FIGURE 4:**
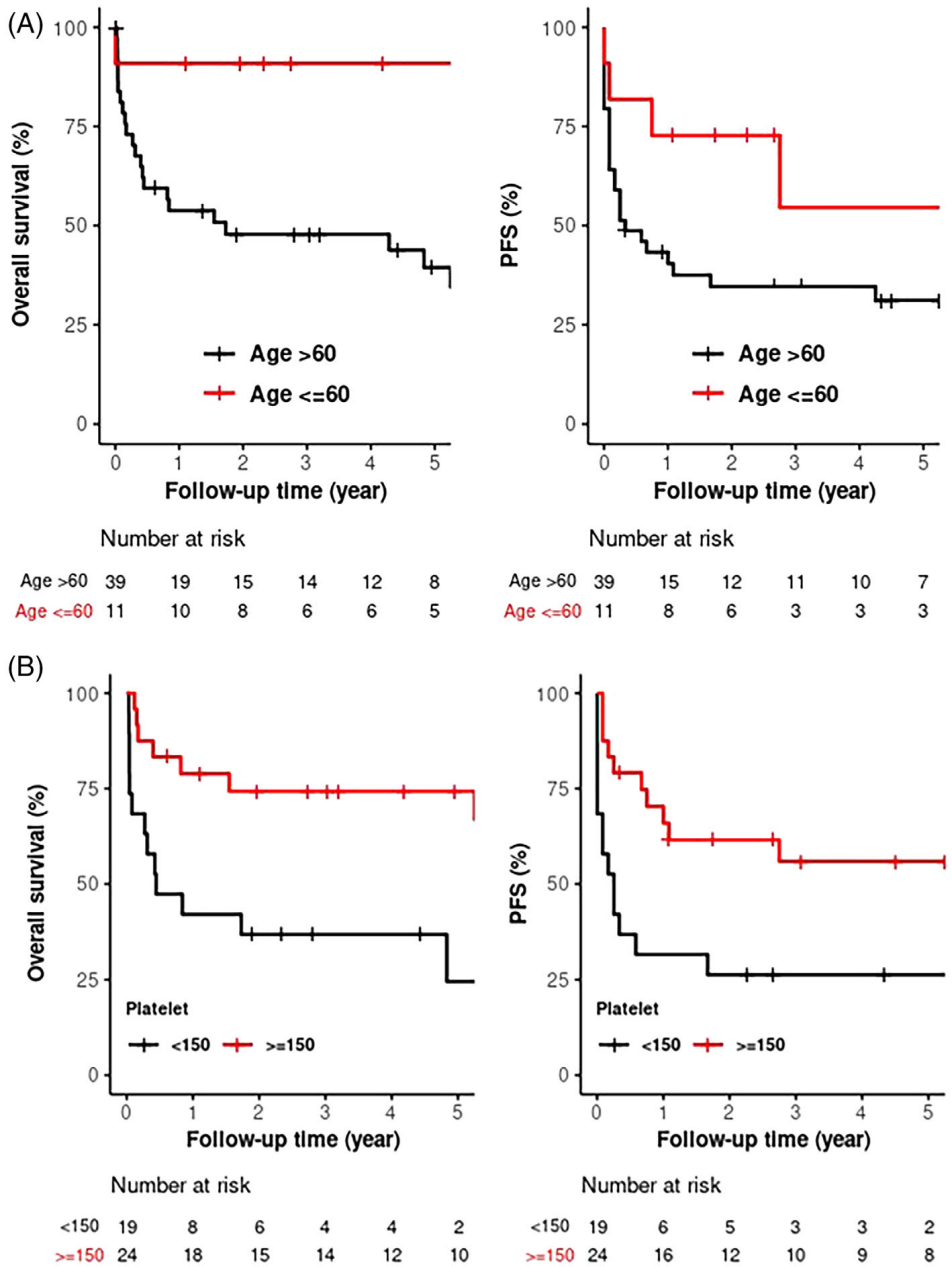
(A) Kaplan–Meier survival curves showing mOS (*p* = .002) and mPFS (*p* = .021) for age ≤ 60 versus age > 60 at diagnosis. (B) Kaplan–Meier survival curves showing mOS (*p* = .005) and mPFS (*p* = .01) for platelet count <150 versus platelet count ≥150 at diagnosis [Color figure can be viewed at wileyonlinelibrary.com]

Total number of patients (N) was 55 with a median follow up of 1.5 years (range: 0–5.1 years), during which 25 patients died. 47% (26/55) of the patients were alive at time of the last follow‐up. Four patients who were diagnosed with CNS‐IVL at autopsy were not included in the survival analysis (Figures 3 and 4, Tables S7‐S9, Figures S1‐S4).

Estimated 5Y progression free survival (PFS) and overall survival (OS) for the entire cohort were 38.6% and 52%. No statistically significant difference in survival was seen between CNS‐IVL and non‐CNS‐IVL. Estimated 5Y PFS and OS were 47.6% and 47.6% (CNS‐IVL) versus 35.2% and 54% (non‐CNS‐IVL), respectively.

Among subtypes of IVL based on anatomic localization on presentation, no significant difference was seen in terms of estimated PFS and OS. Skin‐only IVL appears to be associated with excellent survival. Estimated 5Y PFS and OS were 43.75% and 43.8% (CNS‐only IVL); 53.3% and 100% (skin‐only IVL); 31.82% and 46% (P‐IVL); and 50% and 50% (M‐IVL).

Univariate analysis showed that age > 60Y and platelet count <150 10^9^/L were significant prognostic indicators with negative impact on PFS and OS. Estimated 5‐year PFS and OS was 31.2% and 39.5% (age > 60) versus 60.0% and 91.7% (age < 60) (*p* = .021 and .002); 26.3% and 24.6% (platelet < 150) versus 56% and 74.3% (platelet > 150) (*p* = .01 and .005), respectively. Rituximab containing regimen was associated with significant improvement in outcomes. Estimated 5Y PFS and OS were 50% and 66.8% (rituximab containing regimen) versus 0% and 0% (non‐ rituximab containing regimen), respectively. Gender, LDH, and leucopenia did not have significant impact on the survival outcomes. Multivariate analysis also showed significantly better mPFS and mOS for age less than 60. However, platelet count no longer showed a statistically significant relationship with survival outcomes after adjusting for age maybe due to small sample size and missing platelet data for some patients.

The significance of other variables (HD‐MTX‐based CIT, CNS prophylaxis, ASCT, and cellular lineage) for the survival outcomes cannot be determined due to small numbers and lack of statistical power.

The median overall survival for T‐cell IVL was 123 months [CI 95%, 10‐NR] and (1/5) were alive at time of last follow up. The patient with NK‐cell IVL died around 30 days after presentation despite treatment with Cyclophosphamide/Doxorubicin/Etoposide.

There was no significant difference in PFS and OS with respect to CD5 positivity. Estimated 1Y PFS and OS were 50% and 50% (CD5 positive cases) versus 17% and 50% (CD5 negative cases), respectively.

## 4 DISCUSSION

In our retrospective study, we identified 55 IVL patients over the span of 15 years in a large academic setting indicating that IVL is a rare lymphoma as previously known. In agreement with previous observations, multifocal involvement is most common in our cohort. However, IVL cases clinically confined to the CNS and skin were also seen. Based on anatomic localization and tissue tropism at the time of presentation, our cohort can be categorized into IVL with clinically isolated CNS involvement (CNS‐only IVL) 22%; IVL with mixed clinical CNS and peripheral site involvement (M‐IVL) 14.5%; IVL with clinically isolated skin involvement (Skin‐only IVL) 9%; IVL with peripheral site involvement with or without skin involvement (P‐IVL) 54.5%. The main reason for classifying IVL into CNS‐IVL and non‐CNS‐IVL is that CNS involvement comes with its unique clinical manifestations which tend to be more severe and requires a unique approach to management. For lymphoma in general, assessment of CNS involvement and CNS risk is of paramount importance. The same can be said about IVL. Most importantly, CNS‐IVL is approached differently compared to non‐CNS‐IVL in selection of therapeutic regimen. In our cohort, CNS‐IVL cases were predominantly managed with high‐dose methotrexate (HD‐MTX)‐based chemoimmunotherapy (CIT) whereas non‐CNS‐IVL cases were managed with RCHOP CIT with or without CNS prophylaxis. As such, classifying IVL into CNS versus non‐CNS groups has a practical utility.

CNS involvement did not have significant impact on the survival outcomes in our cohort. However, all four patients diagnosed with IVL at autopsy had CNS involvement and were not included in analysis. No significant difference in survival outcomes was seen among four subgroups. However, skin‐only IVL has excellent survival in line with what has been reported in skin‐only IVL.^6^

IVL has been reported for a protean of clinical manifestations ranging from few symptoms to rapid deterioration with multiorgan failure.^3^, ^13^ The most common reported symptoms include fever, cytopenias, confusion, and stroke‐like symptoms.^3^, ^14^ Our CNS‐IVL patients presented with high incidence of bowel/bladder incontinence, and extremity weakness/gait impairment whereas non‐CNS IVL patients manifested weight loss, skin rash and fatigue as most common presenting features. In our patients with cutaneous involvement, the striking feature is that lower extremities, especially thigh areas are preferentially involved often with bilaterality. Predominant involvement of lower extremities by skin IVL has been previously reported.^15^

The diagnosis of IVL is often missed as indicated by historically high rate of diagnosis at autopsy,^1^, ^7^ with CNS‐IVL having the highest incidence of post‐mortem diagnosis.^6^ Two recent reports indicate post‐mortem diagnosis at 20% and 16%.^8^, ^11^ In our cohort, post‐mortem diagnosis was made in four patients (7.3%) two patients had CNS‐only IVL and 2 with M‐IVL. The incidence of post‐mortem diagnosis can decrease with the increase in awareness of IVL among the clinicians and improvement in diagnostic tools.

MRI and PET imaging scans played an important role in clinching a definitive diagnosis in our cohort. MR showed abnormalities in 85% (17/20) of CNS‐IVL cases. Lymphoma was part of the radiologist's differential diagnosis in few MRI reports (2/17). Further research is needed in this section to further characterize IVL lesions on MRI imaging. 89% (17/19) of our IVL cases with abnormal PET findings had biopsy of a PET‐avid lesion resulting in a diagnosis. As such, our data show for the first time that PET scan plays an important role in diagnosing IVL. The top three biopsy sites in our cohort include bone marrow (45%), brain (29%), and skin (25%). One patient had blind skin biopsies which was diagnostic. Bone marrow was the most frequently diagnostic site in another study.^8^

Pathologically, most of our cases had B‐cell IVL (89%; 48/54). The cohort includes five cases of T‐cell IVL and one case of NK‐cell IVL. All cases with non‐B‐cell IVL had non‐CNS site involvement. MUM1 expression by IHC was seen in 77% of B‐cell IVL cases indicating non‐germinal center cell of origin. We did not find any case of double or triple hit lymphoma in our cohort based on limited testing. Our cohort lacks genomic data such as mutation status of MYD88 and CD79 which were recently found to be present in IVL as frequently as in primary CNS lymphoma.^16^ While the disease is predominantly described as an intravascular process, we highlight that lymphoma cells can also be seen in the parenchyma. This characteristic has previously been described in the literature where IVL can be seen in association with nodal lymphoma as described by Starr, et al.^17^ This phenomenon may be because IVL can acquire the capacity to extravasate and involve the tissue compartment.^4^ The other explanation for the increased incidence of lymph node involvement is that our cohort was evaluated with PET‐CT imaging which might have shown lymph node involvement not detected on CT imaging.

Therapeutically, the most optimal treatment for IVL is not well‐defined due to rarity of lymphoma and lack of prospective clinical trial data. Most frequently, CNS‐IVL cases in our cohort were treated with Rituximab and HD‐MTX‐based regimen whereas non‐CNS‐IVL cases were treated with RCHOP. CNS prophylaxis approaches were also adopted in our cohort. Due to lack of statistical power associated with small numbers, we could not determine the impact of HD‐MTX, CNS prophylaxis, and ASCT on the survival outcomes. The overall survival in our cohort appears to be at least equivalent to the best survival outcome mentioned in the literature. We reported an estimated 5Y OS for the entire cohort of 52%. In contrast, several studies have shown the three‐year overall survival rates ranging between 11.5%–33%; and 5‐year OS in one study was 46%.^8^, ^18^, ^19^, ^20^, ^21^

Our study determined thrombocytopenia as a negative prognostic indicator for IVL. Our statistical analysis showed that platelet count of150 was the best cut off point for predicting both OS and PFS in terms of hazard ratios and p values in our study. In our analysis, 45% (21/47) were thrombocytopenic and appeared to be a high‐risk group as their estimated 5Y OS was only 24.6% compared to 74.3% in non‐thrombocytopenic patients. In agreement with this finding, all four patients diagnosed with IVL at autopsy had thrombocytopenia with three of them having platelet count <20 × 10^9^/L. The underlying mechanism for thrombocytopenia is not clear but is likely related to intravascular localization of IVL which may lead to intravascular destruction of platelets. In this study, 68% (17/25) had ≤20% bone marrow involement which suggests that thrombocytopenia is less likley to be related to a compromised platelet production. Thus, low platelet count may reflect widespread dissemination of IVL. Another poor prognostic indicator identified in this study is age >60 Y. Estimated 5Y OS was 39.5% in the elderly group compared to 91.7% in younger patients. Platelet <100 and age >60 have been previously identified as indicators of poor prognosis.^14^ Novel therapies should be explored for IVL patients with these poor risk factors. However, age was the only significant prognostic indicator on multivariate analysis, likely due to small sample size and missing platelet data for some patients.

CD5 positivity is reported to be associated with higher prevalence of bone marrow involvement, thrombocytopenia and worse survival outcomes,^8^ In contrast, we found CD5 + IVL to be associated with lower prevalence of thrombocytopenia, lower incidence of bone marrow involvement, and no significant difference in survival outcomes compared to CD5‐ IVL. However, the number of CD5+ versus CD5‐ cases were too small in our study to draw any definitive conclusions.

Although this study is one of the biggest studies for IVL, the sample size is still very small for definitive statistical analysis. Due to lack of statistical power, we could not determine the significance of important variables such as role of various therapeutic interventions. In addition, like any other retrospective study, there were different degrees of missing data in the variables of interest, which might have introduced bias in the study

In conclusion, our study shows various interesting findings. Meticulous clinical evaluation inclusive of skin examination with special focus on lower extremities, PET imaging, MRI brain, and bone marrow biopsy is important for timely diagnosis of IVL. Long‐term survival can be achieved in about half of IVL patients with current therapeutic interventions. Thrombocytopenia and old age are negative prognostic indicators for IVL and can be used to identify high‐risk patients. Novel therapeutic approaches are necessary to improve the prognosis of IVL especially in elderly patients. Collaboration across multiple institutions would be necessary to better understand this rare lymphoma and develop novel therapeutic approaches.

## CLINICAL TRIAL REGISTRATION

Not applicable.

## PERMISSION TO REPRODUCE MATERIAL FROM OTHER SOURCES

Not applicable.

## CONFLICT OF INTEREST

The authors declare that the research was conducted in the absence of any commercial or financial relationships that could be construed as a potential conflict of interest.

## Supporting information


**Appendix S1 Figure S1** Kaplan‐Meier survival curves showing mOS (*p* = .54) and mPFS (0.69) by IVL subgroups
**Figure S2** Clinical course with therapeutic outcome events for each patient in the study
**Figure S3** Kaplan‐Meier survival curves showing mOS (*p* = .009) and mPFS (0.001) by if rituximab was received
**Figure S4** Kaplan‐Meier survival curves showing mOS (*p* = .58) and mPFS (0.15) by CD 5 marker
**Table S1** Clinical features at the time of diagnosis and therapeutic interventions
**Table S2** Demographic and baseline clinical characteristics of IVL patients
**Table S3** Skin findings in IVL
**Table S4** Distribution of organ involvement at initial presentation
**Table S5** MRI brain/spine findings in CNS‐IVL
**Table S6** Patients with abnormal PET imaging findings in IVL
**Table S7** Kaplan‐Meier estimates of OS and PFS since diagnosis
**Table S8** Characteristics of CD5 positive and negative cases
**Table S9** Multivariable Cox regression models

## Data Availability

The data that support the findings of this study are available on request from the corresponding author. The data are not publicly available due to privacy restrictions.
