# Supplementary material for: Clinical characteristics, prognostic indicators, and survival outcomes in intravascular lymphoma: Mayo Clinic experience (2003–2018)
Source: Am J Hematol. 2022 Jul 6;97(9):1150–8. doi: 10.1002/ajh.26635 (PMC9541514; doi:10.1002/ajh.26635)
Supplement: Supplementary file 1 — Appendix S1 Figure S1 Kaplan‐Meier survival curves showing mOS (p = .54) and mPFS (0.69) by IVL subgroups Figure S2 Clinical course with therapeutic outcome events for each patient in the study Figure S3 Kaplan‐Meier survival curves showing mOS (p = .009) and mPFS (0.001) by if rituximab was received Figure S4 Kaplan‐Meier survival curves showing mOS (p = .58) and mPFS (0.15) by CD 5 marker Table S1 Clinical features at the time of diagnosis and therapeutic interventions Table S2 Demographic and baseline clinical characteristics of IVL patients Table S3 Skin findings in IVL Table S4 Distribution of organ involvement at initial presentation Table S5 MRI brain/spine findings in CNS‐IVL Table S6 Patients with abnormal PET imaging findings in IVL Table S7 Kaplan‐Meier estimates of OS and PFS since diagnosis Table S8 Characteristics of CD5 positive and negative cases Table S9 Multivariable Cox regression models [file AJH-97-1150-s001.docx]

**Supplementary Figure 1. Kaplan-Meier survival curves showing mOS (p=0.54) and mPFS (0.69) by IVL subgroups**


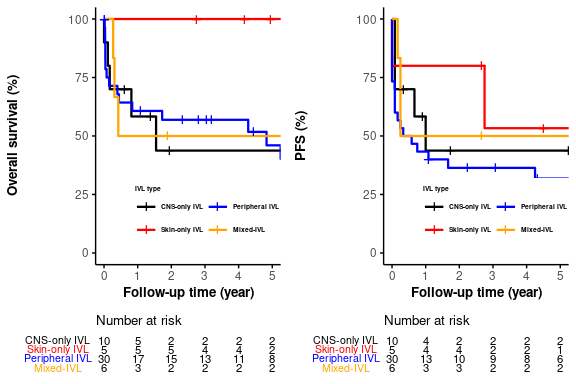


**Supplementary Figure 2. Clinical course with therapeutic outcome events for each patient in the study**


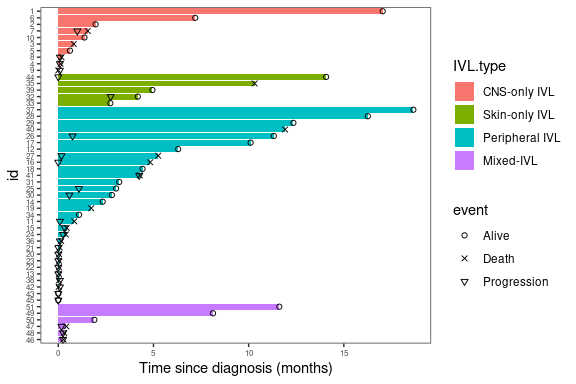


**Supplementary Figure 3. Kaplan-Meier survival curves showing mOS (p=0.009) and mPFS (0.001) by if rituximab was received**


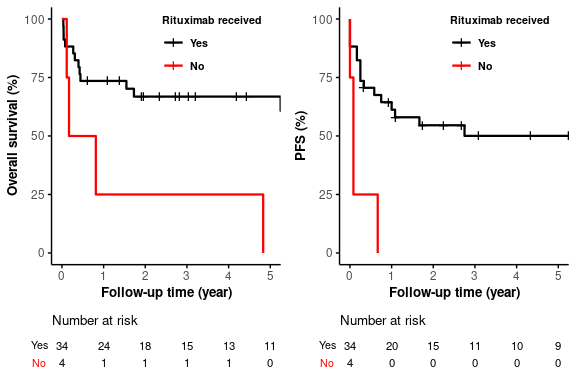


# Supplementary Figure 4. Kaplan-Meier survival curves showing mOS (p=0.58) and mPFS (0.15) by CD 5 marker


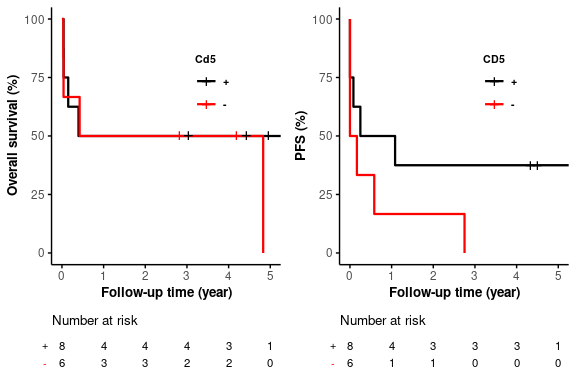


Supplementary Table. 1. Clinical features at the time of diagnosis and therapeutic interventions

|  | | **CNS-IVL** | **Non-CNS-IVL** |
| --- | --- | --- | --- |
| **Symptoms at the time of presentation** | | | |
| **Constitutional** | | | |
| Fatigue | | 26%, (5/19) | 30%, (9/30) |
| Weight loss | | 15%, (3/19) | 40%, (12/30) |
| Loss of appetite | |  | 10%, (3/30) |
| Night sweats | | 25%, (2/8) | 20%, (6/30) |
| Fever | | 38%, (3/8) | 17%, (5/30) |
| **Neurological** | | | |
| Memory disturbances/confusion | | 36%, (7/19) | 10%, (3/30) |
| Visual disturbances | | 21%, (4/19) |  |
| Hearing impairment | | 9%, (1/11) |  |
| Slurred speech | | 10%, (2/19) |  |
| Extremity weakness, or gait impairment | | 42%, (8/19) | 10%, (3/30) |
| Paraesthesia of hands/feet | | 26%, (5/19) | 7%, (2/30) |
| **Gastrointestinal** | | | |
| Nausea/Vomiting | |  | 3%, (1/30) |
| Diarrhea | |  | 3%, (1/30) |
| Abdominal pain | |  | 3%, (1/30) |
| Jaundice | |  | 3%, (1/30) |
| **Hematologic** | | | |
| Lymphadenopathy | |  | 13%, (4/30) |
| Hepatomegaly | |  | 10%, (3/30) |
| Splenomegaly | |  | 50%, (15/30) |
| Easy bruising or bleeding | |  | 10%, (3/30) |
| **Respiratory and cardiovascular** | | | |
| Shortness of breath | | 21%, (4/19) | 20%, (6/30) |
| Chest pain | |  | 3%, (1/30) |
| Pedal edema | |  | 10%, (3/30) |
| **Dermatologic** | | | |
| Skin rash | |  | 43%, (13/30) |
| **Urologic** | | | |
| Back pain | |  | 7%, (2/30) |
| Bowel/bladder incontinence | | 45%, (5/11) | 3%, (1/30) |
| **Therapeutic interventions** | | | |
| Most common first-line regimen | | High dose methotrexate+ Rituximab containing regimen 75%, (12/16) | RCHOP (60%), (17/28) |
| Autologous stem cell transplant | | 21%, (4/19) | 17%, (5/28) |
| CNS prophylaxis for non-CNS IVL | |  | 50%, (14/28) |
| Intrathecal (IT) methotrexate/cytarabine | |  | 14%, (2/14) |
| High dose (HD) methotrexate | |  | 85%, (12/14) |
| CNS relapse for non-CNS IVL | |  | 8%, (3/35) |
| Type of CNS prophylaxis received for those with CNS relapse: | None |  | 1/3 |
|  | HD methotrexate |  | 2/3 |

# Supplementary Table 2. Demographic and baseline clinical characteristics of IVL patients

|  | CNS-IVL (N=20) | Non-CNS-IVL (N=35) | Total (N=55) | P value |
| --- | --- | --- | --- | --- |
| Age at diagnosis |  |  |  | 0.36 |
| Median (Range) | 66.5 (48.0, 78.0) | 68.0 (40.0, 85.0) | 68.0 (40.0, 85.0) |  |
| Mean (SD) | 65.2 (7.8) | 67.1 (10.6) | 66.4 (9.6) |  |
| Gender 0=male; 1=female |  |  |  | 1.00 |
| 0 | 13 (65.0%) | 22 (62.9%) | 35 (63.6%) |  |
| 1 | 7 (35.0%) | 13 (37.1%) | 20 (36.4%) |  |
| ECOG >2 =1 ECOG ≤2 =2 |  |  |  | 0.63 |
| N-Miss (number of patients with missing data for the reported parameter) | 11 | 15 | 26 |  |
| 1 | 2 (22.2%) | 3 (15.0%) | 5 (17.2%) |  |
| 2 | 7 (77.8%) | 17 (85.0%) | 24 (82.8%) |  |
| early diagnosis=1, late diagnosis =2. Earl≤30days. Late>30 days |  |  |  | 1.00 |
| N-Miss | 2 | 6 | 8 |  |
| 1 | 3 (16.7%) | 6 (20.7%) | 9 (19.1%) |  |
| 2 | 15 (83.3%) | 23 (79.3%) | 38 (80.9%) |  |
| Cell type |  |  |  | 0.21 |
| N-Miss | 1 | 0 | 1 |  |
| B cell | 19 (100.0%) | 29 (82.9%) | 48 (88.9%) |  |
| T cell | 0 (0.0%) | 5 (14.3%) | 5 (9.3%) |  |
| NK cell | 0 (0.0%) | 1 (2.9%) | 1 (1.9%) |  |
| BCL6 (IHC) 0=positive, 1=negative |  |  |  | 0.16 |
| N-Miss | 13 | 20 | 33 |  |
| 0 | 6 (85.7%) | 7 (46.7%) | 13 (59.1%) |  |
| 1 | 1 (14.3%) | 8 (53.3%) | 9 (40.9%) |  |
| MYC (IHC) 0=positive, 1=negative |  |  |  | 1.00 |
| N-Miss | 18 | 30 | 48 |  |
| 0 | 2 (100.0%) | 3 (60.0%) | 5 (71.4%) |  |
| 1 | 0 (0.0%) | 2 (40.0%) | 2 (28.6%) |  |
| MUM1 0=positive, 1=negative |  |  |  | 0.62 |
| N-Miss | 12 | 20 | 32 |  |
| 0 | 5 (62.5%) | 12 (80.0%) | 17 (73.9%) |  |
| 1 | 3 (37.5%) | 3 (20.0%) | 6 (26.1%) |  |
| platelet.150 |  |  |  | 0.77 |
| N-Miss | 2 | 6 | 8 |  |
| <150 | 8 (44.4%) | 15 (51.7%) | 23 (48.9%) |  |
| >=150 | 10 (55.6%) | 14 (48.3%) | 24 (51.1%) |  |
| Leukopenic (WCC<3.4 x10^9^ /L) 1=yes, 2=no. |  |  |  | 0.51 |
| N-Miss | 2 | 7 | 9 |  |
| 1 | 4 (22.2%) | 10 (35.7%) | 14 (30.4%) |  |
| 2 | 14 (77.8%) | 18 (64.3%) | 32 (69.6%) |  |
| High ESR (ESR of >22 mm/1 h) 1=yes, 2=no |  |  |  | 1.00 |
| N-Miss | 9 | 21 | 30 |  |
| 1 | 7 (63.6%) | 10 (71.4%) | 17 (68.0%) |  |
| Rituximab containing regimen 1=yes, 2=no |  |  |  | 0.29 |
| N-Miss | 4 | 12 | 16 |  |
| 1 | 13 (81.2%) | 22 (95.7%) | 35 (89.7%) |  |
| 2 | 3 (18.8%) | 1 (4.3%) | 4 (10.3%) |  |
| CD5+=1 CD5-ve =2 |  |  |  | 0.20 |
| N-Miss | 18 | 22 | 40 |  |
| 1 | 0 (0.0%) | 8 (61.5%) | 8 (53.3%) |  |
| 2 | 2 (100.0%) | 5 (38.5%) | 7 (46.7%) |  |

Supplementary Table 3. Skin findings in IVL

| **Patient** | **Location of biopsy** | **Description** |
| --- | --- | --- |
| **Skin-only IVL** | | |
| #1 | bilateral thigh and calf | blue discoloration, induration, lump-like texture, painful, calor |
| #2 | bilateral calf | erythema, peau d'orange appearance of skin, swelling, induration, plaque like features, superficial erosions |
| #3 | bilateral lower extremities | erythema, swelling |
| #4 | bilateral lower extremities | maculopapular rash with psoriatic changes |
| #5 | left thigh | not reported |
| **Peripheral IVL with skin involvement** | | |
| #6 | bilateral thighs and calf | induration, tenderness |
| #7 | upper chest, neck, face (cheeks, scalp), buttocks and thighs | crusted erosions, indurated subcutaneous nodule |
| #8 | inner left thigh | not reported |
| #9 | truck, arms, and legs | diffuse maculopapular erythematous rash |
| #10 | left inner thigh (random skin biopsies) | no rash |
| #11 | left cheek | not reported |
| #12 | right calf | not reported |
| #13 | left calf | not reported |
| #14 | lower abdomen and back | not reported |

# Supplementary Table 4: Distribution of organ involvement at initial presentation

| Organs involved at presentation | Number of patients |
| --- | --- |
| Brain | 18 |
| Spinal cord | 2 |
| Lung parenchyma | 7 |
| Liver | 8 |
| Spleen | 15 |
| Adrenal gland | 3 |
| Prostate gland | 1 |
| Lymph nodes  Cervical  Mediastinal  Axillary  Abdominal  Retroperitoneal  Pelvic  Inguinal | 9 |
|  | 2 |
|  | 4 |
|  | 1 |
|  | 4 |
|  | 4 |
|  | 5 |
|  | 1 |
| Soft tissue of right shoulder | 1 |
| Omentum | 1 |
| Adnexa | 1 |
| Skin | 5 |
| Multi organ involvement | 31 |

Supplementary Table 5. MRI brain/spine findings in CNS-IVL.

| **Patients** | **Location in CNS** | **Localized vs multifocal** | **Infarcts** | **Mass Lesions** |
| --- | --- | --- | --- | --- |
| #1 | Occipital region | Localized | Yes | N/A |
| #2 | Thoracic cord | Multifocal | N/A | N/A |
| #3 | Bilateral cerebral hemispheres and cerebellum | Multifocal | N/A | N/A |
| #4 | Frontal lobes and left frontoparietal region | Multifocal | N/A | N/A |
| #5 | Cerebellopontine angle | Localized | N/A | Yes |
| #6 | Right cerebellum, occipital region, parietal region, leptomeninges | Multifocal | Yes | N/A |
| #7 | Left cerebellar tonsil, left periatrial region, right temporal region, left floccular region | Multifocal | N/A | Yes |
| #8 | Centrum semiovale, bilateral corona radiata | Multifocal | N/A | Yes |
| #9 | Corpus callosum | Multifocal | N/A | N/A |
| #10 | Thoracic cord, central pontine, corpus callosum, right cerebellum | Multifocal | Yes | N/A |
| #11 | Left pons, bilateral frontal region, right parietal, bilateral temporal, regions, leptomeninges | Multifocal | N/A | N/A |
| #12 | Leptomeninges | Localized | N/A | N/A |
| #13 | Left frontal, right parietal, left parietal, leptomeninges | Multifocal | Yes | N/A |
| #14 | Left basal ganglia, left cerebellum | Multifocal | Yes | N/A |
| #15 | Periventricular and centrum semiovale | Multifocal | N/A | N/A |
| #16 | Bilateral hemispheric | Multifocal | N/A | N/A |
| #17 | Right insular cortex, inferior frontal parietal lobe | Localized | N/A | Yes |

**Supplementary Table 6. Patients with abnormal PET imaging findings in IVL**

| **Patient** | **PET findings** | **Cell type** | **PET avid site biopsied** | **Involved sites not avid on PET scan** | **Max SUV reported** |
| --- | --- | --- | --- | --- | --- |
| #1 | Increased FDG avidity of spleen, mediastinal lymph nodes, submandibular lymph nodes, bilateral hilar lymph nodes, and bilateral lung ground-glass lesion | T-cell | Lung lesion | N/A | N/A |
| #2 | Increased FDG avidity right lung nodules, skin of right lower extremity | T-cell | Skin | N/A | 9.7 (skin) |
| #3 | Increased FDG avidity of bone marrow and spleen, (diffuse bone marrow involvement) | NK  cell | Bone marrow | N/A | 13.4 (bone marrow) |
| #4 | Increased FDG avidity of spleen, liver, adrenal gland, bone marrow, (diffuse bone marrow involvement) | B-cell | Bone marrow | N/A | 13.7 (bone marrow) |
| #5 | Increased FDG avidity of liver, spleen, bone marrow, (diffuse bone marrow involvement) | B-cell | Bone marrow | N/A | 7.7 (bone marrow) |
| #6 | Increased FDG avidity of cervical, intrathoracic, intra- abdominal and pelvic lymphadenopathy, bone marrow, (diffuse bone marrow involvement) | B-cell | Lymph node | Skin | N/A |
| #7 | Increased FDG avidity of liver, spleen, bone marrow, (diffuse bone marrow involvement) | B-cell | Bone marrow | N/A | 12.4 (bone marrow) |
| #8 | Increased FDG avidity of bone marrow, (diffuse bone marrow involvement) | B-cell | Bone marrow | N/A | N/A |
| 9# | Increased FDG avidity of skin of bilateral lower extremities | B-cell | Skin | N/A | 2.5  (skin) |
| #10 | Increased FDG avidity of bone marrow of axial and appendicular skeleton, (diffuse bone marrow involvement) | B-cell | Bone marrow | Liver | 3.7  (bone marrow) |
| #11 | Increased FDG avidity of left common iliac lymph nodes, speaking, T10 vertebrae, bone marrow, (diffuse bone marrow involvement) | B-cell | Bone marrow | N/A | N/A |

| #12 | Increased FDG avidity adjacent to abdominal aortic aneurysm graft | B-cell | Soft tissue adjacent to abdominal aortic aneurysm | N/A | N/A |
| --- | --- | --- | --- | --- | --- |
| #13 | Increased FDG avidity of bone marrow, (diffuse bone marrow involvement) | B-cell | Bone marrow | N/A | N/A |
| #14 | Increased FDG avidity of spleen, appendical and axial skeleton (diffuse bone marrow involvement) | B-cell | Bone marrow | N/A | 19.1 (bone marrow) |
| #15 | Increased FDG avidity of right adrenal nodule | B-cell | Adrenal gland | Prostate | 7.6  (adrenal gland) |
| #16 | Increased FDG avidity nodule in anterior mediastinum | B-cell |  | Skin | 2.8 |
| #17 | Increased FDG avidity of soft tissue nodules over the right chest wall. | B-cell | Skin | N/A | 6.4  (skin) |
| #18 | Increased FDG avidity lymph nodes in neck, chest, abdomen, pelvis, spleen, left ocular region | B-cell |  | Bone marrow | 12.5 (lymph node) |
| #19 | Increased FDG avidity supraclavicular lymph nodes, mediastinal lymph node, lung nodule, abdominal lymph nodes | B-cell | Mediastinal lymph node, lung biopsy | N/A | N/A |

# Supplementary Table 7. Kaplan-Meier estimates of OS and PFS since diagnosis

| **Term** | **Total** | **Total Events** | **1-year freedom from event** | **3-year freedom from event** | **5-year freedom from event** | **P value** |
| --- | --- | --- | --- | --- | --- | --- |
| **Overall survival** | | | | | | |
| All | 51 | 25 | 63.2% (51.0%, 78.3%) | 58.7% (46.3%, 74.4%) | 52.0% (38.8%, 69.5%) | - |
| Site: 1= any CNS IVL; 2= vs non-CNS IVL only |  |  |  |  |  | 0.81 |
| 1 | 16 | 8 | 55.6% (35.6%, 86.63%) | 47.6% (27.8%, 81.51%) | 47.6% (27.8%, 81.51%) | - |
| 2 | 35 | 17 | 66.7% (52.4%, 84.86%) | 63.5% (49.0%, 82.31%) | 54.0% (38.4%, 76.15%) | - |
| Site: 1=CNS-only IVL, 2=Skin-only IVL, 3=P-IVL, 4= M-IVL |  |  |  |  |  | 0.54 |
| 1 | 10 | 5 | 58.3% (34.0%, 100.0%) | 43.8% (20.0%, 95.7%) | 43.8% (20.0%, 95.7%) | - |
| 2 | 5 | 1 | 100.0% (100.0%, 100.0%) | 100.0% (100.0%, 100.0%) | 100.0% (100.0%, 100.0%) | - |
| 3 | 30 | 16 | 60.7% (45.1%, 81.8%) | 56.9% (41.2%, 78.7%) | 46.0% (29.6%, 71.4%) | - |
| 4 | 6 | 3 | 50.0% (22.5%, 100.0%) | 50.0% (22.5%, 100.0%) | 50.0% (22.5%, 100.0%) | - |
| Age >60 1=yes, 2=no |  |  |  |  |  | 0.002 |
| 1 | 39 | 24 | 53.8% (39.9%, 72.6%) | 47.8% (34.0%, 67.3%) | 39.5% (25.6%, 60.9%) | - |
| 2 | 12 | 1 | 91.7% (77.3%, 100.0%) | 91.7% (77.3%, 100.0%) | 91.7% (77.3%, 100.0%) | - |
| Gender 0=male; 1=female |  |  |  |  |  | 0.20 |
| 0 | 32 | 18 | 57.8% (42.7%, 78.2%) | 54.2% (39.0%, 75.2%) | 49.2% (33.8%, 71.8%) | - |
| 1 | 19 | 7 | 72.2% (54.2%, 96.2%) | 66.2% (47.4%, 92.4%) | 56.7% (36.2%, 89.0%) | - |
| platelet.150 |  |  |  |  |  | 0.005 |
| <150 | 19 | 13 | 42.1% (24.9%, 71.338%) | 36.8% (20.4%, 66.378%) | 24.6% (9.1%, 66.326%) | - |
| >=150 | 24 | 8 | 78.9% (64.1%, 97.196%) | 74.3% (58.5%, 94.412%) | 74.3% (58.5%, 94.412%) | - |
| Leukopenic 1=yes, 2=no |  |  |  |  |  | 0.090 |
| 1 | 13 | 4 | 76.9% (57.1%, 100.0%) | 76.9% (57.1%, 100.0%) | 76.9% (57.1%, 100.0%) | - |
| 2 | 29 | 16 | 58.4% (42.9%, 79.5%) | 50.6% (35.1%, 73.1%) | 43.4% (27.0%, 69.8%) | - |
| Rituximab containing regimen 1=yes, 2=no |  |  |  |  |  | 0.009 |
| 1 | 34 | 12 | 73.5% (60.1%, 90.0%) | 66.8% (52.5%, 85.1%) | 66.8% (52.5%, 85.1%) | - |
| 2 | 4 | 4 | 25.0% (4.6%, 100.0%) | 25.0% (4.6%, 100.0%) | 0.0% (NA, NA) | - |
| CD5+=1 CD5-ve =2 |  |  |  |  |  | 0.58 |
| 1 | 8 | 4 | 50% (25.0%, 99.980%) | 50% (25.0%, 99.980%) | 50% (25.0%, 99.980%) | - |
| 2 | 6 | 4 | 50% (22.5%, 100.000%) | 50% (22.5%, 100.000%) | 0% (NA, NA) | - |
| **Progression-free survival** | | | | | | |
| All | 51 | 32 | 48.7% (36.6%, 64.6%) | 41.6% (29.7%, 58.2%) | 38.6% (26.7%, 55.7%) | - |
| Site: 1= any CNS IVL; 2= vs non-CNS IVL only |  |  |  |  |  | 0.33 |
| 1 | 16 | 8 | 47.62% (27.8%, 81.5%) | 47.62% (27.8%, 81.5%) | 47.62% (27.8%, 81.5%) | - |
| 2 | 35 | 24 | 48.57% (34.5%, 68.3%) | 39.11% (25.7%, 59.6%) | 35.20% (22.0%, 56.3%) | - |
| Site: 1=CNS-only IVL, 2=Skin-only IVL, 3=P-IVL, 4= M-IVL |  |  |  |  |  | 0.69 |
| 1 | 10 | 5 | 43.75% (20.00%, 95.7%) | 43.75% (20.00%, 95.7%) | 43.75% (20.00%, 95.7%) | - |
| 2 | 5 | 3 | 80.00% (51.61%, 100.0%) | 53.33% (21.42%, 100.0%) | 53.33% (21.42%, 100.0%) | - |
| 3 | 30 | 21 | 43.33% (28.78%, 65.2%) | 36.36% (22.58%, 58.6%) | 31.82% (18.47%, 54.8%) | - |
| 4 | 6 | 3 | 50.00% (22.46%, 100.0%) | 50.00% (22.46%, 100.0%) | 50.00% (22.46%, 100.0%) | - |
| Age >60 1=yes, 2=no |  |  |  |  |  | 0.021 |
| 1 | 39 | 28 | 40.4% (27.5%, 59.4%) | 34.6% (22.3%, 53.8%) | 31.2% (19.2%, 50.7%) | - |
| 2 | 12 | 4 | 75.0% (54.1%, 100.0%) | 60.0% (34.7%, 100.0%) | 60.0% (34.7%, 100.0%) | - |
| Gender 0=male; 1=female |  |  |  |  |  | 0.48 |
| 0 | 32 | 21 | 39.8% (25.887%, 61.3%) | 39.8% (25.887%, 61.3%) | 39.8% (25.887%, 61.3%) | - |
| 1 | 19 | 11 | 63.2% (44.800%, 89.0%) | 44.3% (25.922%, 75.7%) | 36.9% (19.380%, 70.3%) | - |
| platelet.150 |  |  |  |  |  | 0.010 |
| <150 | 19 | 14 | 31.6% (16.3%, 61.2%) | 26.3% (12.4%, 55.8%) | 26.3% (12.4%, 55.8%) | - |
| >=150 | 24 | 11 | 66.0% (49.3%, 88.3%) | 56.0% (38.6%, 81.2%) | 56.0% (38.6%, 81.2%) | - |
| Leukopenic 1=yes, 2=no |  |  |  |  |  | 0.31 |
| 1 | 13 | 6 | 61.5% (40.04%, 94.6%) | 53.8% (32.55%, 89.1%) | 53.8% (32.55%, 89.1%) | - |
| 2 | 29 | 18 | 47.6% (32.38%, 70.1%) | 38.8% (23.99%, 62.8%) | 38.8% (23.99%, 62.8%) | - |
| Rituximab containing regimen 1=yes, 2=no |  |  |  |  |  | 0.001 |
| 1 | 34 | 16 | 61% (47%, 80.2%) | 50% (35%, 71.5%) | 50% (35%, 71.5%) | - |
| 2 | 4 | 4 | 0% (NA, NA) | 0% (NA, NA) | 0% (NA, NA) | - |
| CD5+=1 CD5-ve =2 |  |  |  |  |  | 0.15 |
| 1 | 8 | 5 | 50% (25.0%, 99.98%) | 38% (15.3%, 91.74%) | 38% (15.3%, 91.74%) | - |
| 2 | 6 | 6 | 17% (2.8%, 99.74%) | 0% (NA, NA) | 0% (NA, NA) | - |

# Table 8. Characteristics of CD5 positive and negative cases

| **Variables** | **CD5 positive** | **CD5 negative** |
| --- | --- | --- |
| Entire cohort | 53% (8/15) patients | 46% (7/15) patients |
| Whites | 6/8 | 7/7 |
| Blacks | 1/8 |  |
| Asian | 1/8 |  |
| Males | 4/8 | 4/7 |
| Age>60 | 8/8 | 6/7 |
| Median Age at diagnosis | 72 years (range 67-82) | 71 years (range 58-85) |
| ECOG >2 at diagnosis | 1/5 | 1/5 |
| Mean Hb at diagnosis | 9.8g/dL (range 1.7-16) | 8.7 g/dL (range 7-12) |
| Anemia | 7/1 | 7/7 |
| Mean white cell count at diagnosis | 6x10^9^ /L (range (3-11) | 4.5 x10^9^ /L (range (2-7.1) |
| Leukopenic | 2/7 | 1/7 |
| Mean platelet count at diagnosis | 140 x10^9^ /L (range 25-266) | 65 x10^9^ /L (range 6-296) |
| Thrombocytopenia at diagnosis | 4/8 | 6/7 |
| Mean ESR at diagnosis | 36 mm/1 h (range 1-103) | 71 mm/1 h (range 40-116) |
| LDH > upper limit of normal | 5/6 | 6/7 |
| Mean LDH | 729 U/L (range 217-1391) | 866 U/L (range 208-1822) |
| B-cell IVL cases | 6/8 | 6 |
| T-cell IVL cases | 2/8 |  |
| NK cell IVL cases |  | 1 |
| Hepatomegaly | 0/8 | 2/5 |
| Splenomegaly | 4/8 | 4/7 |
| M-IVL |  | 2/7 |
| P-IVL | 7/8 | 4/7 |
| Skin-only involvement | 1/8 | 1/7 |
| Multiorgan involvement | 6/8 | 6/7 |
| Median duration of onset of symptoms to biopsy proven diagnosis IVL only in days | 90 days (range 14-240) | 81 days (range 7-360) |
| Median time between presentation to health care provider and diagnosis in days | 5 days (range 1-157) | 13 days (range 2-74) |
| Abnormal PET | 5/6 | 5/7 |
| Diffuse bone marrow uptake on PET | 3/4 | 3/4 |
| Biopsy of PET avid lesion leading to diagnosis | 5/5 | 3/5 |
| Bone marrow involved | 4/6 | 6/7 |
| ≥20% bone marrow involvement | 2/4 | 4/7 |
| Anthracycline based chemotherapy | 5/8 | 6/7 |
| mOS, p=0.41 | 4 months (1-NR) | 0 months (0-NR) |
| mPFS, p=0.11 | 8 months (1-NR) | 1 month (0-NR) |

# Supplementary Table 9. Multivariable Cox regression models

| **Term** | **HR (95%CI)** | **P value** |
| --- | --- | --- |
| **Model predicting overall survival** |  |  |
| Age >60 | 16.34 (2,11, 2103.59) | 0.003 |
| Platelet <150 | 2.12 (0.88, 5.51) | 0.093 |
|  |  |  |
| **Model predicting progression free survival** |  |  |
| Age >60 | 4.6 ( 1.04 , 20.4 ) | 0.045 |
| Platelet <150 | 2.02 ( 0.88 , 4.67 ) | 0.099 |
